# Human Umbilical Vein Endothelial Cells Express the DUX4 Protein: A Basis for Further Vascular Research

**DOI:** 10.5146/tjpath.2025.14362

**Published:** 2025-09-30

**Authors:** Ceren Hangul, Dilek Bahar, Özlem Ekinci, Öznur Tokta, Yusuf Ozkul, Sibel Berker Karauzum

**Affiliations:** Department of Medical Biology and Genetics, Akdeniz University, Faculty of Medicine, Antalya, Türkiye; Betul-Ziya Eren Genome and Stem Cell Center (GENKOK), Erciyes University, Kayseri, Türkiye; Department of Medical Genetics, Nigde Ömer Halisdemir University, Niğde, Türkiye; Department of Medical Genetics, Erciyes University, Faculty of Medicine, Kayseri, Türkiye

**Keywords:** DUX4, Human umbilical vein endothelial cells, HUVEC, PAX3, PAX7, Cancer, Dystrophy, FSHD

## Abstract

***Objective: *** A growing body of evidence suggests a correlation between endothelial cell dysfunction and cancer, as well as facioscapulohumeral dystrophy, both of which are *DUX4*-related diseases. However, the endogenous expression of *DUX4* within endothelial cells (ECs) remains unexplored. This study aims to investigate DUX4 expression in ECs and examine the presence of DUX4-counteracting proteins named PAX3 and PAX7.

***Material and Methods:*** This is a cell study in which human umbilical vein endothelial cells (HUVECs) were selected as an in vitro representative of the EC population. The presence of the DUX4, PAX3 and PAX7 proteins in the HUVECs was examined using immunofluorescence staining. The mRNA levels of these proteins were investigated using qPCR with specific primers for each transcript.

***Results: *** It was observed that 51% of HUVECs expressed the DUX4 protein whereas only a small number of cells were stained with PAX3/PAX7 antibody. At the mRNA level, HUVECs exhibited expression of *DUX4*, *PAX3* and *PAX7*. The mRNA levels of *PAX3* and *DUX4* were lower compared to *PAX7* mRNA.

***Conclusion:*** The high rate of DUX4 protein expression observed in HUVECs is the first positive data and suggests a potential role for DUX4 protein in endothelial cells. Further analyses including the functional analyses of DUX4, PAX3 and PAX7 in ECs could improve our understanding of a vascular pathogenesis in DUX4-related diseases, particularly in the contexts of cancer and facioscapulohumeral dystrophy.

## INTRODUCTION

Endothelial cells (ECs) have a pivotal role in the pathophysiology of some types of diseases such as cancer and dystrophy. Despite the apparent divergence of these two types of disease, there is an overlapping protein named Double Homeobox 4 (DUX4). The *DUX4* gene is located in the terminal region of the q arm of chromosome 4 (1). Under normal conditions, the DUX4 protein is expressed in 2-4 cell stages of the embryonic period (2,3), and some types of somatic cells e.g., keratinocytes (4), thymus cells (5) and lymphoblast cells (6). Endogenous DUX4 expression in ECs has not been revealed yet. The present study aims to address this gap by investigating endogenous DUX4 expression in ECs.

Angiogenesis is one of the most critical factors in tumour formation and progression. The endothelial cell plays a fundamental role in this process (7), and dysfunctional endothelial cells have been shown to stimulate inflammation and metastasis in cancer (8). Recent studies have demonstrated that DUX4 is prominently expressed in more than 20 types of cancer (9), including leukaemia (10) and sarcoma (11).

ECs have also important functions related with skeletal muscle. It has been demonstrated that certain types of EC progenitors are capable of supporting muscle cells or, alternatively, directly differentiating into skeletal muscles, a process termed myo-endothelial progenitor differentiation (12). One of the most prevalent dystrophies, facioscapulohumeral dystrophy (FSHD), manifests as a consequence of the deletion of D4Z4 repeat containing *DUX4* gene, and FSHD is accompanied by impaired endothelial cell signature (13). Furthermore, there is evidence from studies of FSHD cases that retinal vascular abnormalities (14), thrombocyte number changes (15) and renal endothelial anomalies (16) support a critical link with endothelial function. However, there is a paucity of data on DUX4 expression in endothelial cells in FSHD.

All these aforementioned overlapping findings on DUX4 with (i) endothelial cell, (ii) cancer and (iii) dystrophy directed us to hypothesise that DUX4 can be an active key transcriptional factor in endothelial cells.

Various DUX4 isoforms are synthesized by alternative splicing. The DUX4s isoform, of which the carboxyl terminus (C) is absent, contains only the N-terminal region (N), which facilitates binding to the target gene. The C terminal region has been demonstrated to be necessary for target gene activation (17). The primary form capable of initiating transcriptional activation by binding to the target gene is defined as a transcription factor (TF) in the literature. This form is referred to as the full-length (DUX4-fl) isoform, which contains the C-terminal region. As the objective of this study was to ascertain the fundamental active form, detailed analyses were conducted on the full-length DUX4.

Pioneer transcription factors play a pivotal role in the initiation of cell differentiation and the subsequent activation of cell-specific genes. Recent evidence suggests that DUX4 functions as a pioneer transcription factor in mammals (2). The two key pioneer transcription factors PAX3 and PAX7 were found to be functionally exchangeable with DUX4 protein. Cells expressing PAX3 and PAX7 have been revealed to have the ability to intervene with the effects of DUX4 (18). In addition, PAX3 has been identified to be critical as a transcription factor in the determination of cell fate, particularly in circumstances where cells must decide to differentiate into endothelial cells or a muscle cells. Furthermore, the expression of PAX3 directed mesenchymal cells (MSCs) of the bone marrow (BM) to differentiate into myogenic lineage (19). PAX7 is another key factor to understand the functions of DUX4, because PAX7 has been shown to be an equivalent biomarker showing DUX4 target gene expression (20). For these reasons, PAX3 and PAX7 were integrated in the present study to facilitate a more comprehensive investigation.

In summary, the present study aims to investigate the presence of DUX4 and the expression patterns of PAX3 and PAX7 in ECs. Recent developments in single-cell RNA sequencing (scRNA-seq) have made it possible to uncover previously unrecognized endothelial cell heterogeneity (21). In order to achieve these objectives *in vitro*, Human Umbilical Vein Endothelial Cells (HUVECs) have been selected as a model system for the function and pathology of ECs. This hypothesis is supported by data acquired regarding the active chromatin mark of DUX4 detected in HUVECs (22). This study presents the first data on the existence of DUX4 protein in HUVEC samples, in conjunction with PAX3 and PAX7 expression.

## MATERIAL and METHODS

The aim of this study was to determine whether DUX4, PAX3, and PAX7 are expressed in HUVEC cells. In line with this objective, mRNA levels were investigated through RT-PCR analysis with specific primers following the extraction of RNA from HUVEC cells. Protein presence was investigated by immunohistochemistry using specific antibodies. This study had been approved by the Akdeniz University Medical Scientific Research Ethics Committee (Approval No: TBAEK-561)

### Cell Culture

HUVECs were obtained from the American Type Culture Collection (ATCC) company and cell culture experiments were performed at the Genomics and Stem Cell Centre. Cells were cultivated in Dulbecco’s Modified Eagle Medium (DMEM) containing 20% foetal calf serum (FBS), 1% penicillin-streptomycin, and 1% L-Glutamine within a T25cm2 flask at 37 °C humidified CO2 incubator. Cells that had reached 85-90% confluency were then washed with Dulbecco’s Phosphate Buffer saline (dPBS). Thereafter, cells were detached using trypsin-EDTA and subsequently subjected to centrifugation at 300xg for 5 minutes. The cell pellet was cultured in a T75cm2 flask until an adequate number of cells was attained. Residual cells were stored in liquid nitrogen for subsequent studies.

### RNA extraction

The cells were washed twice with dPBS and 1 ml of TRIzol was added, the solution was incubated for five minutes. Subsequently, the solution was vortexed. Then 200 µl of chloroform was added and vortexed for an additional 15 seconds. The cells were then centrifuged at 12,000xg for 15 minutes at room temperature. The aqueous phase was transferred to a new tube and 500 µl isopropanol was added. The mixture was then incubated at -10 °C for 10 minutes. Subsequently, the sample was centrifuged at +4 °C. The cell pellet was resuspended in 75% ethanol and centrifuged once more at 7,500 x g for five minutes at 4 °C. The resulting pellet was then left to dry, and the pellet was resuspended in DNase and RNase water.

### cDNA synthesis

The total RNA was converted into cDNA using a Blue-Ray PCR device in accordance with the protocol set out in the Applied Biosystems High-Capacity cDNA Reverse Transcription Kit (4368814). The conditions for the reverse transcriptase reaction were as follows: 25 °C for 10 minutes, 37 °C for 120 minutes, 85 °C for 5 minutes, and 4 °C. The cDNA samples were stored at -20 °C.

### Spectrophotometric Measurement of cDNA Samples

The quantity and purity of the isolated cDNA samples were measured using a spectrophotometer (DENOVIX // DS11 FX+). For the measurement, a 1 μl volume of cDNA sample was loaded into the spectrophotometer. The purity and quantity of the cDNA were determined in ng/μl. The ratio of measurements was obtained at the 260/230 nm and 280/260 nm wavelengths. cDNA samples were diluted to 50 ng/μl for the subsequent PCR experiments.

### Quantitative Real-Time Polymerase Chain Reaction (qRT-PCR)

The LONGGENE-Q2000B Real-Time PCR device and hibrigen 2X SYBR Green qPCR Mix kit (MG-SYBR-01-80) were used in accordance with the protocol specified to determine the levels of *DUX4, PAX7*, and *PAX3 *gene expressions. Mixes containing the cDNAs of HUVEC samples were added to strip tubes with optical caps including negative control containing no cDNA. *B-actin* was used as the housekeeping reference gene. The average of the results is calculated and integrated into the graph. The primer sequences used for the *DUX4, PAX3, PAX7* and *B-actin *were given in Table 1 *.* qPCR conditions were: 95 °C 1 minutes; and 40 cycles for 95 °C 15 seconds, 60 °C for 15 seconds, 72 °C for 45 seconds. Amplicon specificity was verified by melt curve analysis. Each experiment was conducted using three independent biological replicates with a minimum of two technical replicates. The qPCR values thus obtained were then plotted as the average of the replicate experiments.

**Table 1:** Forward and reverse primer sequences of *DUX4, PAX7, PAX3* and *B-actin* genes

***Gene***	***Sequence***	***Sequence***
	**Forward**	**Reverse**
DUX4	5’-CAAGGGGTGCTTGCGCCACCCACGT-3’	5’-GGGGTGCGCACTGCGCGCAGGT-3’
PAX7	5’- AACCACATCCGCCACAAGATA-3’	5’-GCCTGGGTTTTCCCTCTTGTA-3’
PAX3	5’- GGAGACTGGCTCCATACGTC-3’	5’- CAAATTACTCAAGGACGCGG-3’
B actin	5’-CCTGGCACCCAGCACAAT-3’	5’-GCCGATCCACACGGAGTACT-3’

### Immunocytochemistry

The cells were seeded onto 8 well chamber plates and incubated for 24 hours until they had attached. The attached cells were fixed by incubation in 4% paraformaldehyde for five minutes at room temperature. Subsequently, the cells were washed three times with dPBS, and cells were permeabilized with 0.3% Triton X-100 for three minutes at room temperature. Then the cells were washed again and blocked with 1% IgG-free bovine serum albumin (BSA) at room temperature to prevent non-specific bonding. Then the cells were washed again, and primary antibodies DUX4 (#AB229810, Abcam) and PAX3/PAX7 (#sc365843, Santa Cruz) added at a dilution of 1:100 in dPBS and incubated overnight at +4°C (PAX3 and PAX7 proteins have multiple isoforms and an antibody binding both of these proteins and their isoforms were used). The following day, the cells were washed once more, and secondary antibodies were added at a dilution of 1:500 at room temperature for 1 hour. The antibodies used were mouse antibody Fitch, #A21202 and rabbit Texas Red, #21207 (Life Tech). Then cells were washed and DAPI containing mounting medium was added. Imaging was conducted by using a fluorescence microscope (Nikon Ti eclipse 100). Control images for the immunofluorescence experiments have been included as a supplementary figure (Supplementary Figure 1).

## RESULTS

In view of the evidence indicating a role in endothelial cell dysfunction, DUX4, PAX3 and PAX7 were investigated at the mRNA and protein levels in HUVECs.

### The Expression of DUX4, PAX3 and PAX7 mRNAs is Positive in HUVECs

**Figure 1:**
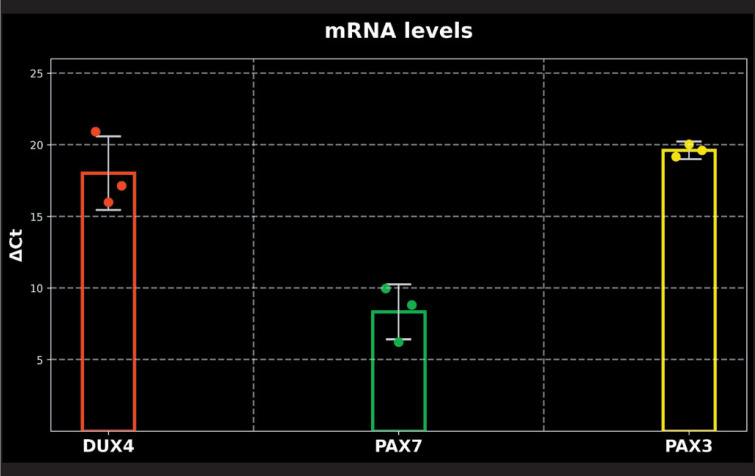
Relative mRNA levels of DUX4 (left), PAX7 (middle), and PAX3 (right) normalized to B actin, presented as ΔCt values determined by qPCR. Bars represent the mean ± standard deviation (SD). No statistically significant difference was observed among the genes (one-way ANOVA, p=0.3). qPCR analysis was performed using three independent biological replicates, each measured with a minimum of two technical replicates.

The presence of DUX4, PAX3, and PAX7 had not previously been detected in HUVECs. The primary objective of the present study was to ascertain whether expression was present at the mRNA level. The results demonstrated that the *DUX4*, *PAX3* and *PAX7* mRNAs were expressed in HUVEC samples (Figure 1). Of particular note was the finding that *PAX7* mRNA levels were significantly higher compared to those of DUX4 and PAX3 mRNAs. Furthermore, *PAX7* expression exhibited a peak at the 19th cycle (Ct Mean: 19.48 ± 1.9), while *DUX4* expression exhibited a peak at the 29th cycle (Ct Mean: 29.16 ± 2.5) and *PAX3* expression peaked at the 32nd cycle (Ct Mean: 32.89 ± 0.6) in the qPCR analysis. Statistical comparison of gene expression levels, normalised to the housekeeping gene B-actin and analysed using ΔCt values, revealed no significant differences among *DUX4*, *PAX7*, and *PAX3* mRNAs.

### Marked DUX4 Protein Staining was Observed to be Localised in the Nucleus of HUVECs

The subsequent phase of the study was to investigate whether the existing *DUX4*,* PAX3*, and *PAX7* mRNAs were translated into protein. Examination of immunofluorescence analyses revealed markedly positive DUX4 staining.

**Figure 2:**
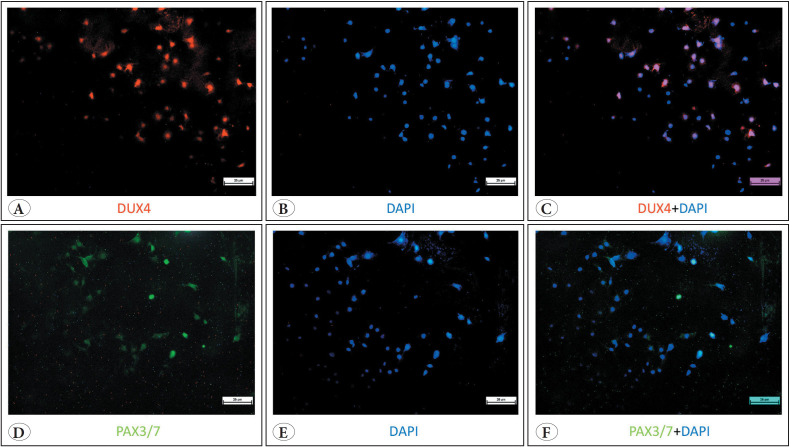
Immunofluorescence analysis of DUX4 and PAX3/PAX7 in HUVECs separately. Nuclei were counterstained with DAPI (blue). **A)** DUX4 (green) protein staining **B)** DAPI (blue) staining. **C)** DUX4+DAPI merged image. **D)** PAX3/PAX7 (green) protein staining **E)** DAPI (blue) staining. **F)** PAX3/PAX7 + DAPI merged image.

Immunofluorescence-based analysis of microscopic fields revealed 142 out of 280 cells were DUX4-positive (Supplementary Figure 2) corresponding to an average proportion of 51% with a standard deviation of 35%. Wilson score intervals were calculated for each field to provide robust confidence estimates of binomial proportions. The mean of the lower bounds of the Wilson confidence intervals across fields was 42.6%, while the mean of the upper bounds was 59.1%. No statistically significant differences were observed in the number of DUX4-positive cells (p = 0.07). In order to specify the cellular localisation of the signal, DAPI staining was applied. Following DAPI merge, it became evident that the majority of the DUX4 were located within the nucleus. Additionally, a minor degree of cytoplasmic DUX4 expression was observed. A more detailed analysis of the nuclear staining pattern revealed a range of nuclear staining patterns among the cells, rather than a single standardised pattern: some cells exhibited complete nuclear staining, while others displayed a variable degree of nuclear staining (Figure 2). PAX3 & PAX7 protein staining was observed only in few cells with a very low level of detection. The signals belonging to PAX3 and PAX7 proteins were observed in the nuclear region (Figure 2, Supplementary Figure 3).

### Co-expression of PAX3&PAX7 with DUX4 was a Rare Event

**Figure 3:**
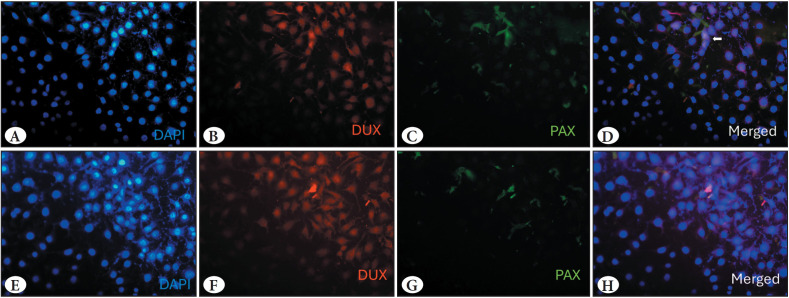
Merged immunofluorescence analysis of DUX4 and PAX3/PAX7 co-expression in HUVECs. Nuclei were counterstained with DAPI (blue) **A,E)** DAPI (blue) staining **B,F)** DUX4 (red) protein staining. **C,G)** PAX3/PAX7 (green) staining **D,H)** Merged images of DUX4 and PAX3/PAX7 staining.

PAX3 and PAX7 are shown to act in competition with DUX4. Haynes *et al* revealed that PAX3 and PAX7 couldn’t be observed together in the same cell with DUX4 protein because they are spatially distinct in stem cells (23): if DUX4 is present PAX3 or PAX7 would not be expressed and vice versa. Compatible with previous literature findings, we revealed that DUX4 and PAX3 & PAX7 protein expressions were localised in different cells (Figure 3). In contrast to previous reports, we qualitatively observed co-expression of DUX4 and PAX7 in this study (Figure 3, *white arrow*). Based on this observation, the frequency of double-positive nuclei was assessed across eight distinct regions, revealing that 5 out of 185 cells were double-positive (Supplementary Figure 3).

## DISCUSSION

DUX4 is a pioneer transcription factor that can activate a number of target genes. DUX4 is expressed during critical time periods including the embryonic period (2), and in different cell types such as keratinocytes (4), thymus (5), testicular (24) and bone marrow (25) samples. There are still numerous cell types in which endogenous DUX4 expression has yet to be investigated. Despite the increasing evidence for the disturbance of endothelial function in DUX4-related diseases such as cancer and dystrophy, the endogenous DUX4 expression in endothelial cells (ECs) has not been investigated previously. A recent study revealed the presence of histone 3 lysine 27 acetylation (H3K27ac) small peaks in HUVECs, indicating an active chromatin mark associated with DUX4 expression (22). This finding indicates that HUVECs are an appropriate endothelial cell model for investigating DUX4 expression. The present study constitutes an investigation into the presence of endogenous DUX4 expression and its counteracting proteins PAX3 and PAX7 in HUVECs *in vitro* for the first time.

The analysis of mRNA via qPCR revealed the presence of endogenous *DUX4, PAX3* and *PAX7* mRNAs in HUVECs with different levels of expression. *PAX7* was the most prominent mRNA, followed by *DUX4, *with *PAX3 *mRNA exhibiting the lowest levels. The observed low level of *PAX3* mRNA in this sample may suggest that the HUVECs possess a structure that differed from the muscle cells yet maintain similarities with the myogenic lineage, as *PAX3* had not completely disappeared.

Despite the absence of research examining endogenous DUX4 expression in human ECs, there is evidence of exogenous DUX4 expression in ECs that locates in muscle tissue. A study conducted using the iDUXpA-HSA mice model identified the consequences of DUX4 on muscle tissue subsequent to transgenic transfer (26). The dystrophic mice exhibited vascular structure formation disorders, including capillary reduction. Furthermore, DUX4-expressing mice exhibited a reduction in EC population (26).

A study of single cell transcriptomics revealed that muscle stem cells (MuSCs) express prototypic markers of ECs (27). MuSCs are stem cells that facilitate the regeneration of adult skeletal muscle. MuSCs and ECs arise from a common bipotent ancestor (28). PAX3 was found to be capable of differentiating these ancestors towards the myogenic line (19). Together with PAX3 mRNA expression, DUX4 expression in HUVECs could be further investigated for the cell fate of the progenitors.

A review of the transcriptomic studies conducted on DUX4-related cancers and dystrophies reveals a significant amount of data supporting the hypothesis that there could be a common pathway regarding endothelial function related with DUX4 and PAX3 in cancer in addition to dystrophy. The existing literature contains findings from a variety of studies that support this hypothesis. The fusion of *PAX3* with *FOXO* has been demonstrated to effectively reprogram human and mouse human endothelial precursors resulting in the transformation of these cells to rhabdomyosarcoma (29). It is noteworthy that DUX4 is already expressed endogenously in rhabdomyoma cell lines (30).

A very recent study revealed a case of DUX4-positive endothelial neoplasm. A detailed investigation into RNA research in cancerous tissue revealed the presence of a *CIC::DUX4* fusion. Furthermore, DUX4 immunochemistry revealed a high level of DUX4 protein. The methylation testing of the tumour indicated a diagnosis of angiosarcoma rather than CIC-rearranged sarcoma (31). This is a very striking finding suggesting that DUX4 could serve as a valuable target for cancer treatment via angiogenesis. The identification of the role of DUX4 in angiogenesis in the cancer niche is a topic that merits further research in the future.

In a study investigating the lymphatic metastasis of prostate cancer, markers expressed in co-cultivation with endothelial cells were identified; *DUX4 *was one of the identified factors followed by *HIF1* and *MATR3* (32). A more thorough examination of the functions of these proteins reveals that HIF1 is a pivotal protein in ECs related to angiogenesis, and that MATR3 is a recently identified therapeutic target for a dystrophy that is strictly related to DUX4 (33). This dystrophy is known as Facioscapulohumeral Muscular Dystrophy (FSHD). The disease is caused by a deletion of the DUX4 locus. Almost all the information we know about the DUX4 protein is derived from studies that have been conducted on FSHD.

A substantial body of both direct and indirect evidence in FSHD, supports a link between DUX4 and the vascular pathogenesis of FSHD. Abnormal arterial structure (tortuosity) has been observed on fundus examination in a number of FSHD cases and this vascular abnormality correlates with the severity of the disease (14,34). Furthermore, FSHD has been associated with a decrease in endothelial cells (35), dysregulated endothelial transcriptome (36), and VEGF-A signalling (37). Levels of VEGF-A were found to be lower in the serum of FSHD patients and correlated with muscle weakness (38). VEFG-A signalling is the key factor that recruits ECs to muscle and improves cell survival of MuSCs *in vivo* and* in vitro* (27).

The results obtained demonstrated that the DUX4 protein was predominantly positive in the majority of the HUVECs, whereas PAX3/PAX7 protein staining was identified in a limited number of cells (Figure 3, Supplementary Figure 3). This result is similar to the finding that the majority of nuclei in myotube cells were positive for DUX4 protein, while only a few cells were observed to express DUX4 mRNA in skeletal cells (39).

In HUVECs, quantification of 280 cells across 10 representative images revealed that 142 cells were DUX4-positive, highlighting the substantial proportion of cells expressing DUX4 and supporting its potential role in the observed cellular phenotype. An average 51% DUX4 protein expression among microscopic fields are consistent with previous transcriptomic analyses reporting heterogeneous DUX4 expression, in which two distinct populations of DUX4-affected nuclei could be defined by their transcriptional profiles (40).

We also observed that the mRNA expression levels of *DUX4* and *PAX3* were lower in comparison to *PAX7*. *PAX7* exhibited an earlier proliferation peak, as evidenced by a lower deltaCT value (Figure 2). However, at the protein level, DUX4 was found to have the highest expression. This result can be explained by a previous finding, which revealed that DUX4 expression inhibited PAX3/PAX7 accumulation by posttranscriptional regulation in skeletal muscles (41). It is hypothesised that the DUX4 level itself might be diminishing PAX7 protein level in HUVECs. Further functional studies incorporating a more comprehensive approach including protein stability and half-life differences will be valuable in evaluating this suggestion.

Upon examination of the cellular localisation of DUX4 signals, it was observed that there was a notable presence within the nucleus, with less cytoplasmic distribution (Figure 2). The results were consistent with Kowaljow’s muscle data in which DUX4 was shown to be predominantly expressed in the nuclear region (30). In the study conducted by Chau *et al*., the cellular location of the DUX4 protein was investigated including both endogenous and recombinant forms (39). It was stated that cytoplasmic signals belonged to recombinant DUX4 and were present in cells with intense nuclear staining. In the present study, cytoplasmic DUX4 signals were observed in the cells exhibiting higher nuclear expression. However, these signals belonged to the endogenous form of DUX4 (Figure 2).

It is notable that the DUX4 staining pattern observed in the nucleus exhibits variation among individual cells. In Figure 2, approximately 25% (16/65) of the cells exhibited DUX4 staining, with the majority displaying prominent nuclear staining, while the remainder exhibited less pronounced nuclear staining of variable intensity. A comparable phenomenon is observed in Chau’s study, where greater staining was observed for the full-length DUX4. Our result may also suggest distinct cell populations in HUVECs exhibiting low or high DUX4 expression. This variable expression merits further investigation.

As a pioneer transcription factor, DUX4 is capable of initiating a series of interconnected changes within the cell. The DUX4 exerts its influence by binding to the promoter regions of target genes. These binding sites are also the regions where the PAX3 and PAX7 transcription factors can bind and compete with DUX4. The current hypothesis is that endogenous DUX4, PAX3 and PAX7 proteins do not naturally coexist within a cell. A study conducted on the myogenic differentiation process with a 40-day differentiation process. As a result, PAX3 and PAX7 expression along with DUX4 was not observed in any of the cells (23). In our study, the majority of cells exhibited DUX4 and PAX3/PAX7 expressions in distinct cells. Interestingly, co-expression of DUX4 and PAX3/7 was detected only in a small fraction of cells (5/185). Although this finding was unexpected, it may point to a context-dependent or transient interaction between the two factors and the existence of different DUX4-related mechanisms in ECs compared to muscle cells. Further analyses, including quantitative co-localisation and mechanistic studies, are needed to validate and better understand this potential association.

The high level of DUX4 protein expression in HUVECs suggests the presence of key connections and transformations between endothelial cells with cancer cells and with skeletal muscle cells. The expression of DUX4 protein at the appropriate time and in the optimal quantity may be a contributing factor in the initiation of vascular angiogenesis. This may represent a crucial point for further investigation to understand the etiology of DUX4-related diseases. Conducting future studies in this manner has the potential to lead to ground-breaking treatment options for cancer and muscular dystrophies.

## Limitations of the Study

The present study examined the presence of DUX4 expression in human endothelial cells, thereby demonstrating the expression of the gene for the first time. These results were obtained using human cell lines. In order to reach definitive conclusions, further studies are required in tissue-specific endothelial human cells. These studies will shed light on the endothelial-related pathophysiological conditions.

## Availability of Data

The data that support the findings of this study are available from the corresponding author upon reasonable request.

## Conflict of Interest

The authors declare no conflict of interest.  

## Funding

This study was financially supported by the local University Scientific Research Foundation.

## Ethical Approval

This study had been approved by the Akdeniz University Medical Scientific Research Ethics Committee (Approval No: TBAEK-561).
